# Dynamics of Visual Adaptation With Simultaneous Stimulation of Two Visual Pathways

**DOI:** 10.3389/fnins.2021.719499

**Published:** 2021-08-23

**Authors:** Clemente Paz-Filgueira, Michael Tan, Sarah Elliott, Dingcai Cao

**Affiliations:** ^1^Visual Perception Laboratory, Department of Ophthalmology and Visual Sciences, University of Illinois at Chicago, Chicago, IL, United States; ^2^Department of Bioengineering, University of Illinois at Chicago, Chicago, IL, United States; ^3^Peppermill Resort Spa Casino, Reno, NV, United States

**Keywords:** color vision, adaptation, visual pathways, color higher-order mechanisms, psychophysics

## Abstract

Primates’ retinal ganglion cells in different visual pathways have been shown to adapt independently (Current Biology 22 (2012) 220–224). However, the manner in which adaptation occurs under simultaneous stimulation of two visual pathways has not yet been explored. In this study, the dynamics of color afterimages were measured while stimulating one or two visual pathway using a time-varying afterimage paradigm. The dynamics of adaptation was approximately equivalent among the three primary visual pathways, but adaptation was slower for simultaneous stimulation of two visual pathways compared to the stimulation of one visual pathway. In addition, we found that the speed of adaptation also depends upon which two pathways are combined. We developed a two-stage adaptation model, both with the same dynamics, to account for the results with simultaneous stimulation of two pathways.

## Introduction

**FIGURE 1:**
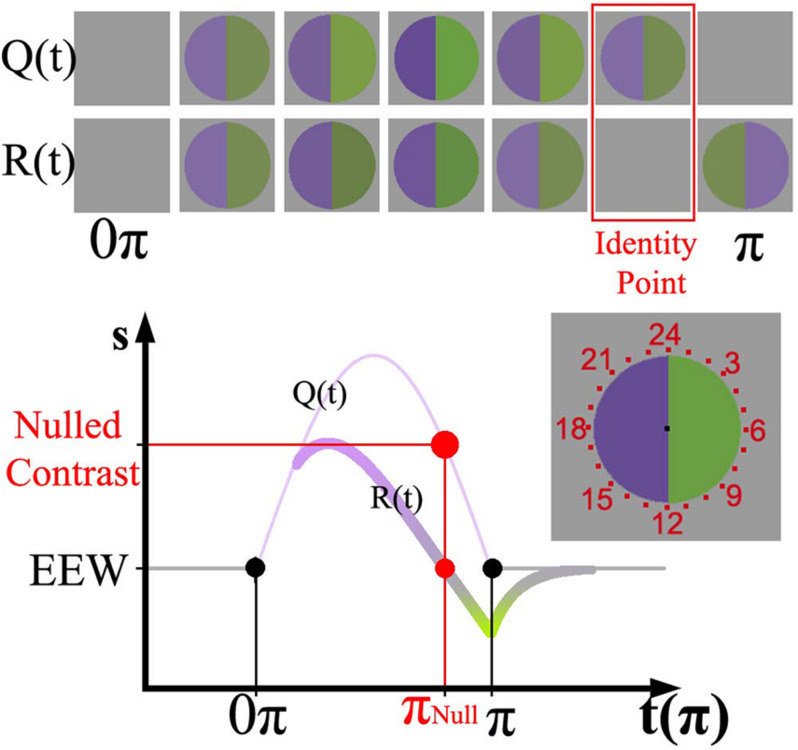
(Top) representation of Zaidi’s paradigm [Q(t)] and the perceptual response over time [R(t)]. (Bottom) sinusoid intensities modulated in the violet hemifield and physiological response curve for RGCs painted according to the perceptual response. The relative amplitude of the sinusoid with respect to the baseline determines the instantaneous contrast of the stimulus. The nulling or identity point is highlighted in red in both representations. The stimulus was surrounded by evenly spaced numbers from 1 to 24 (at the right of the Cartesian representation); in the graph just a set of those numbers is shown.

Visual adaptation refers to a sensitivity change following exposure to a visual stimulus. Adaptation can be fast (in tens and hundreds of milliseconds) (Victor, 1987; Baccus and Meister, 2002) or slow (in seconds or more) (Smirnakis et al., 1997; Chander and Chichilnisky, 2001; Baccus and Meister, 2002). Slow adaptation is closely related to visual afterimages, a lingering perceptual effect experienced after fixating on a steady stimulus that is then removed. Many studies have used afterimages to understand the mechanisms of adaptation (Burbeck and Kelly, 1984; Burbeck, 1986; Kelly and Martinez-Uriegas, 1993). In a more recent report using afterimages to study adaptation, Zaidi et al. (2012) devised a temporally varying stimulus to induce adaptation and evoke afterimages. By modulating with half a sinusoid the intensities of two hemifields from an equal energy white background to opposite ends of a cardinal color axis, they found that the perceptual response reached “gray” before the physical stimulus returned to an equal energy white background. The upper part of Figure 1 shows a representation of both the physical appearance of the stimulus Q(t) and the perceptual response R(t) over time. They designed a way to precisely record the time where the perceptual “gray” null occurred. In addition, they performed *in vivo* recordings of primates’ retinal ganglion cells (RGCs) in response to their stimulus. Remarkably, they found that primate physiology and human psychophysics followed the same adaptation curve. The RGC responses were explained by a mathematical model which considered a fast mechanism responding directly to the stimulus and a slow, longer-lasting decay process accounting for adaptation. This method is therefore useful to psychophysically study the dynamics of adaptation in different visual pathways.

Visual information is conveyed from the retina to the brain by three primary visual pathways, including: the magnocellular (MC-), parvocellular (PC-), and koniocellular (KC-) pathways. The results from Zaidi et al. (2012) suggested that adaptation is largely driven by retinal mechanisms. However, they used stimulus conditions that stimulated only one visual pathway at a time. It is unclear how processes of adaptation operate when two visual pathways are stimulated simultaneously and higher order chromatic mechanisms may be involved (Webster and Mollon, 1994). Therefore, the primary goal of the current study is to measure adaptation by using the time varying paradigm (Zaidi et al., 2012) with chromaticities that vary along the cardinal and non-cardinal axes. An extension of Zaidi et al. model of two-stage adaptation is proposed to account for our results.

## Materials and Methods

### Observers

Three males (age: 30, 33, and 49 years) and one female (age: 20 years) participated in the study. They all had normal color vision as assessed with the Oculus HMC Anomaloskop (Oculus Optikgeräte GmbH, Germany). The study protocol was approved by the Institutional Review Board of the University of Illinois at Chicago and was in accordance with the Declaration of Helsinki. Informed consent was obtained from all participants.

### Apparatus

We used a calibrated NEC 17-inch CRT monitor for stimulus presentation. The CRT calibration included measurement of spectral outputs of the red, green and blue guns with a Photo Research PR-670 Spectrophotometer (Photoresearch, Chatsworth, CA). Each gun was linearized using measurements of 1024 light levels from an International Light Radiometer/Photometer (IL-1700). The MATLAB Psychophysics Toolbox (Brainard, 1997; Pelli, 1997; Kleiner et al., 2007) was used for stimulus presentation and data collection. For each observer, a heterochromatic flicker photometry (15 Hz) procedure was used to establish equiluminance between the CRT guns.

### Stimuli

Color stimuli were controlled to target each visual pathway, as well as combinations of two of them. The stimuli consisted of two semi-circle hemifields, which together formed a circle subtending 3.6 degrees with a mean luminance of 20cd/m^2^, at such a luminance rod intrusion should be minimal (Curcio et al., 1990; Stockman and Sharpe, 2006; Zele and Cao, 2015).

The time-varying afterimage paradigm (Zaidi et al., 2012) was used to measure adaptation via a temporally linked variable. The lights in the hemifields were modulated with a half cycle of a sinusoid function (16 s, 1/32 Hz) to opposite ends of a color axis, such that the modulation started and finished at zero contrast (baseline). Figure 1 shows the sinusoid stimulus function [Q(t)] and the associated physiological response function, R(t). Whereas the physical stimulus Q(t) is always positive respect to the baseline, R(t) presents positive and negative values separated by what is called the identity point (marked in red). The identity point is linked to an instantaneous stimulus contrast (nulled contrast) and time (nulled π), marked in red on the y and x axes of Figure 1, respectively. The full extension of the stimulus is here referred to as π, and 0.5π corresponds to the moment the stimulus reaches the peak of the sinusoid. Perceptually, the identity point is identified when both hemifields appear the same as the background. The top of Figure 1 shows a graphical representation of the physical stimulus and the corresponding perceptual appearance at various time points. Sequentially, the percept starts after stimulus onset and reaches gray (i.e., the identity point) before the physical stimulus reaches zero contrast. Immediately after the identity point, a complementary color (i.e., afterimage) is experienced.

The two hemifields were centered on the screen and surrounded by 24 radial evenly distributed numbers, resembling an analog clock. After 10.15 s, a red clock hand appeared (9 min of arc thick) going from the center of the stimulus to a randomly selected number displayed on the screen. The clock hand sequentially pointed to one number of the clock face until the end of the stimulus, remaining for about 240 ms in one position before moving to the next one. The task of the observers was to report the time (i.e., number on the clock face) at which both hemifields appeared the same as background (i.e., where the identity point occurred).

**FIGURE 2:**
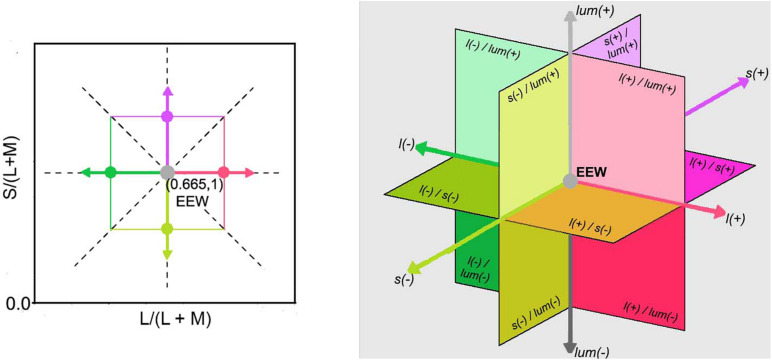
(Left) Representation of the McLeod & Boynton cone chromaticity space showing cardinal and non-cardinal axes on the equiluminant l-s plane with the L/(L + M) axis set as reference, instead of M/(L + M). The two dashed line diagonals in the graph represent two non-cardinal axes, one with positive slope (same polarity) and the other with negative slope (opposite polarity). The angles of them with respect to the horizontal or vertical cardinals are illustrative and do not necessarily match the angles used in this experiment. (Right) A three-dimensional Cartesian representation with a luminance axis perpendicular to an equiluminant plane and origin in EEW is shown. The directions of increments [l(+), s(+), and lum(+)] and decrements [l(–), s(–), and lum(–)] for each cardinal axis are specified as well as the regions where combinations of two channels are stimulated.

**FIGURE 3:**
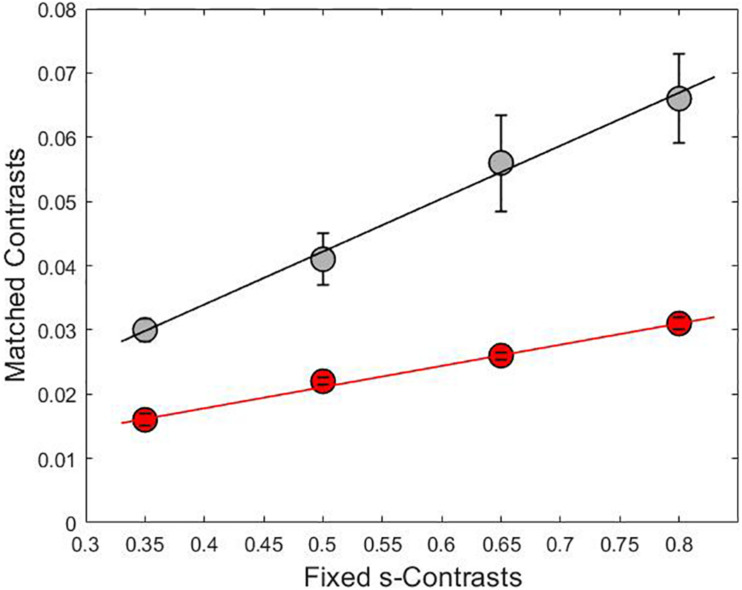
The relationship between the adjusted contrasts on lum-axis (gray line) and l-axis (red line) versus fixed contrasts on s-axis with linear fit lines. The slopes for each line were 0.08 for the gray curve and 0.03 for the red curve and the coefficient of determination was *R*^2^ = 0.99 in both cases. Each point is the result of averaging the matched contrasts of four subjects.

Chromaticities were specified in a modified l, s cone-based chromaticity space (MacLeod and Boynton, 1979) such that the unit of s = S/(L + M) was normalized to 1 for an equal energy spectrum “white” (EEW) light. The l- coordinate [l = L/(L + M)] for this EEW light was 0.665. We added a third axis perpendicular (Figure 2) to the isoluminant plane and representing luminance (L + M + S), so three planes were defined for our stimuli: the isoluminant plane S/(L + M) vs. L/(L + M) and two planes that modulated both luminance and color: L + M + S vs. S/(L + M) and L + M + S vs. L/(L + M). All sinusoidal modulations started and ended on a uniform EEW background of 20cd/m^2^. Stimuli were modulated along one of the three cardinal axes [luminance = (L + M + S), l = L/(L + M) and s = S/(L + M)] or along the non-cardinal axes (i.e., modulation was along two axes simultaneously with the same or opposite polarity). Non-cardinal same polarity stimuli are referred by using a positive sign, for example “lum&s(+)” means modulation of both the luminance and s-coordinates with the same polarity. On the other hand, “lum&s(–)” means modulation with opposite polarity for lum- and s-coordinates. Therefore, there were in total 9 modulation conditions (3 cardinal and 6 non-cardinal axes). We tested four contrasts along each axis. A contrast matching method was used to establish equality of the perceived contrasts for sinusoidal modulations (4 Hz) in different cardinal axes and those contrasts were also used for modulation in intermediate axes. As the s-axis had the highest threshold, we used the s-axis as the reference axis in contrast matching. The s-Weber contrasts were 0.35, 0.50, 0.65, and 0.80. The s-modulation (sinusoidal chromaticities from bluish to yellowish at 4 Hz) was presented randomly in one of the hemifields, and the l- or luminance modulation (reddish/greenish and lighter/darker gray at 4 Hz, respectively) was presented in the other hemifield. Observers adjusted the *l*- or luminance contrast until the perceived contrasts in both hemifields appeared the same. Observers repeated contrast matching 5 times for each s-contrast. All observers consistently showed linearity between the matching contrasts versus s-contrast (see Figure 3 for the averaged matching contrast values of all four observers). The obtained matching luminance or *l*-contrasts for each observer were used for the main experiment.

For each observer, we measured the contrast detection threshold for a 400ms pulse in each axis using a 2-yes-1-no staircase procedure. The averaged contrast detection threshold for the four observers was 1.0% ± 0.3% in the luminance axis, 0.12% ± 0.04% in the l-axis and 3.5% ± 2.0% in the s-axis. Our results were similar to what reported by Wuerger et al. (2020): 1.73% (Weber contrast) for achromatic and 0.26% for red-green.

### Procedure

Observers were seated in front of the monitor inside a dark room with a chin-rest to stabilize the position of the head. The distance from eyes to the monitor was set at 57cm. All measurements were binocular.

Observers first adapted to the uniform EEW background for 1 min. before the experiment began. For each trial, observers reported the time to reach identity point (i.e., both hemifields appeared gray) by typing a whole number from 1 to 24 on a keyboard and then pressing “Enter” to finish the recording and start a new trial. After each trial, the uniform EEW background was presented while the program waited for the observer to press a key to start the next trial. One session consisted of 108 trials (9 axes × 4 contrasts × 3 repetitions) and lasted approximately 50 min. All observers performed 10 sessions on 10 different days.

## Results

**FIGURE 4:**
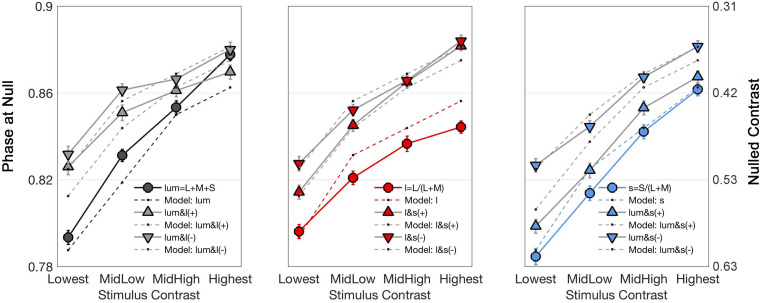
Curves showing the time (in terms of π, left axis) and contrast (percentage of the maximal stimulus contrast, right axis) at which the identity point was reached for the average of all observers as a function of stimulus contrasts. Each graph shows the responses to one cardinal and to two non-cardinal stimulation: lum-axis and stimuli in the lum/l plane (left), l-axis and stimuli in the l/s plane (center), and s-axis and stimuli in the lum/s plane (right). In each graph, circle markers correspond to data from cardinal stimulation while triangle markers correspond to data from the same polarity (labeled as “ + ”) modulation and inverted triangles for the data from the opposite polarity (labeled as “–”) modulation. Solid lines connect data points while dashed lines are model fits. Note that there is a flattening effect at high contrasts, therefore the lowest contrast is taken for analysis.

Figure 4 shows the stimulus phase at null (denoted as nulled π) and nulled contrasts for modulations along the cardinal and intermediate non-cardinal axes. Each panel contains the averaged identity point over 30 trials from the four observers combined along one cardinal axis (l-, s- or lum-) and two non-cardinal axes. Nulled contrast on the right y-axis was normalized as the percentage of the maximal stimulus contrast (corresponding to the peak of the sinusoid). There was a positive relationship between the occurrence of the identity point and stimulus contrast (linear regression analysis, Pearson *r* = 0.84), although some curves in Figure 4 suggest that the relation is not necessarily linear. All identity point contrasts were higher than the contrast detection thresholds found for each observer (*t*-test, α = 0.05) meaning that the point at which they identified a uniform background was shorter than the time they would have perceived a uniform background in the absence of an afterimage.

**FIGURE 5:**
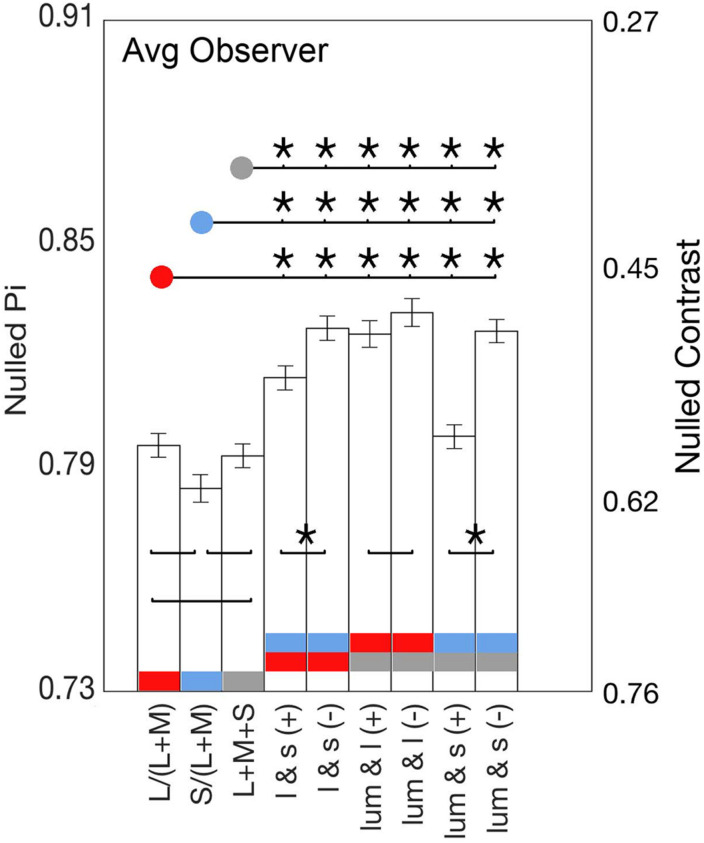
Bars show the average identity points for the lowest contrasts of all nine axes. Significant Fisher comparisons between conditions (*p* < 0.05) are connected with a line and marked with an asterisk (*); those that lack an asterisk were not statistically significant (*p* > 0.05). Comparisons between cardinals and between positive slope (same polarity) and negative slope axes (opposite polarity) are over the bars, whereas comparisons between cardinal and non-cardinal axes are above the bars.

Figure 4 shows that the average phase at null for the non-cardinal axes (represented by triangles for the same polarity modulation and inverted triangles for the opposite polarity modulation) was higher than for cardinal axes (represented by circles), i.e., it took longer to reach the identity point for the non-cardinal axes compared to cardinal axes. It can also be seen that curves representing the non-cardinal axes with same polarity modulation (up-pointing triangles) are below curves corresponding to intermediate axes with opposite polarity modulation (down-pointing triangles). When this analysis performed only on the lowest contrast (where matched contrast variability is minimal, see Figure 3) showed that generally modulation with the same polarity has a lower phase than modulation with the opposite polarity. Figure 5 shows the average results from the four observers for the lowest contrast. First, it confirmed the observation that stimulation along the non-cardinal axes showed a consistent increase in nulled time compared with the cardinal stimulations (Fisher comparisons, α = 0.05), and that modulations with the same polarity had significantly lower nulled time than modulations with the opposite polarity except for the luminance with l-combinations where no statistical difference was found. This analysis also showed no significant differences in the identity points for stimuli presented along the cardinal directions (either lum- vs. l-, lum- vs. s- or l- vs. s-), suggesting when contrasts were equated, the three cardinal axes had comparable dynamics of adaptation.

### Adaptation Model

The physiology-based adaptation model of Zaidi et al. (2012) describes primates’ RGC responses to the time varying paradigm. It proposes that the signal that carries the direct response to the stimulus feeds into a parallel process where an adaptation signal is accumulated and then provides a negative feedback to the original signal. However, the previous model cannot account for the response to simultaneous stimulation of two visual pathways found in the current psychophysical experiments. Here, the physiologically based model of Zaidi et al. is extended by incorporating two-stage adaptation to account for the psychophysical behavior found for cardinal, same polarity non-cardinal and opposite polarity non-cardinal responses.

**FIGURE 6:**
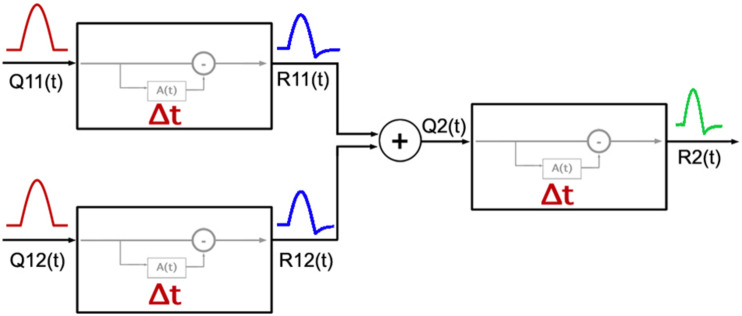
Diagram of the model adopted for the response of the visual system to cardinal and non-cardinal stimulation with a time varying paradigm. The first two units on the left represent two types of RGCs, each with a cumulative function A(t) as modeled by Zaidi et al. (2012) with its own Δ parameter setting the timing properties of adaptation in each visual pathway. Responses from the first stage [R_11_(t) and R_12_(t)] are pooled, and then used as the input [Q_2_(t)] for the second stage mechanism.

Figure 6 shows a diagram for the two-stage model. The first stage corresponds to RGCs and the second stage pools signals from multiple pathways in the first stage. The pooling mechanism in the second stage likely occurs in the cortex as the visual pathways from the retina to LGN are parallel.

We assumed that the second stage has a similar adaptation process to the first stage. The adaptation signal in each stage is modeled using the same equations as proposed by the Zaidi and colleagues:

(1) R(t)=R0+Q(t)-κ*A(t);ν

(2) $$A\,\left(t\right)=\left[A\,\left(t-\Delta \,t\right)+\omega^{*}\,Q\,\left(t-\Delta \,t\right)\right].\exp\,\left(-\Delta \,t/\tau \right);$$

where R(t) is the output of each unit, Q(t) represents the input signal and A(t) is the adaptation signal. The adaptation signal A(t) depends directly from Q(t) and follows a cumulative process, which is subject to an exponential decay (with a time constant τ). Δt determines how quickly the signal A(t) is out of phase with respect to R(t). Parameters κ and ν are scaling factors.

The current model considers the same values for parameters ω, κ, and ν in each stage. The parameter τ was fixed at 8 s for all stages. According to physiological recordings in RGCs, most of the cells studied had values between 5 and 12 s, whereas using a psychophysical setup, Kelly et al. (Kelly and Martinez-Uriegas, 1993) found similar time decay constants for afterimages elicited by achromatic and chromatic stimuli. As we used contrast matching to equate stimulus contrasts from different pathways, we assume that all three pathways have the same input magnitudes at the beginning of the first stage. Then, signals from two pathways in the first stage are combined through a Quick Pooling mechanism in the second stage (Quick, 1974; Sun et al., 2001; Meyer et al., 2005):

(3) Q(t)2=nR(t)11+nR(t)12;n

where R_11_ and R_12_ are outputs from the two pathways in the first stage, Q_2_ is the pooled response arising from the combination of R_11_ and R_12_ and serves as the input for the second stage, and n is the pooling parameter.

Regarding the responses for non-cardinal stimulation, we consistently found shorter responses to same polarity stimulus group [lum&s(+), lum&l(+), and l&s(+)] compared to opposite polarity stimulus group [lum&s(–), lum&l(–), and l&s(–)]. Based on these results, we believe there might be some differences in the processing of signals with same or opposite polarity. We modeled these distinct non-cardinal behaviors by fitting one n parameter for all conditions sharing the same polarity characteristic [denoted as n_(+__)_] and another n parameter for all conditions sharing the opposite polarity characteristic [n_(__–__)_]. Finally, the parameter Δt was set to have the same value in all three units in the first stage (representing magno-, parvo-, and konio-pathways), but a different value was searched for the second stage. Therefore, there are four parameters for each observer in the model, n [n_(__+__)_ or n_(__–__)_], and Δt for each stage. Table 1 shows the fitted parameters for each individual. The small RSS suggested the model is sufficient to account for the data. All Δts for the first stage ranged between 0.18 and 0.25 s, and fitted Δts for the second stage are 3 to 4 times larger than those for the first stage. The pooling parameter n ranged between 0.4 and 1.8 for stimulation with the same polarity and they were in all cases higher than the n parameter fitted for stimulation with the opposite polarity (ranging between 0.2 and 0.6).

**TABLE 1:** Parameters used to fit the model to the responses of the four subjects who performed the experiment and the average of them.

	ω = 0.8, κ = 0.04, ν = 0.6, τs = 8				
	Δt (Sec) Stage 1	Δt (Sec) Stage 2	n _(+)_	n _(__–__)_	RSS
Avg	0.23	0.69	0.8	0.4	0.0017
S1	0.18	0.59	0.6	0.4	0.0046
S2	0.23	0.82	0.4	0.2	0.0037
S3	0.25	0.8	0.7	0.6	0.0087
S4	0.22	0.77	1.8	0.5	0.0094

*Parameters ω, κ, ν, and τ were fixed for all stages and subjects while parameter Δt of the first and second stage as well as the pooling parameter n [n_(+)_ for same polarity and n_(__–__)_ for opposite polarity] were fitted individually to each subject response. The residual sums of squares (RSS) are also presented.*

## Discussion

In this work, the dynamics of adaptation of each of the three visual pathways as well as adaptation when two visual pathways are stimulated simultaneously were assessed. It was found that dynamics of adaptation depend directly on the contrast of the stimulus regardless of which pathway or the number of pathways being stimulated. It was also identified that the dynamics of adaptation among the separate visual pathways are similar when stimulus contrasts are matched, but non-cardinal stimulation yielded different adaptation behavior compared with cardinal stimulations.

The analysis using the lowest contrast showed that temporal properties of adaptation appear to be approximately equivalent in MC-, PC-, and KC-pathways. This trend was not consistent among the four contrasts tested, specially for the two highest contrasts where identity points in the luminance axis resulted in slower adaptation compared to the *l*- and *s*-axis causing the lum-curve to cross over the non-cardinal curves (Figure 4). In turn, the identity points in the *l*-axis were faster, flattening the curve away from the intermediate axes. In this study we used a red clock hand (9 min of arc thick and chromaticities *l* = 0.825, *s* = 0.075, luminance = 20 cd/m^2^) which remained for about 240 ms in one position of the clock face. Given the chromatic and luminance characteristics of the clock hand, it should interfere with the target stimulating mainly in the parvo-cellular pathway. However, the size and duration at one position of the clock hand would minimize that parvo-effect. By making the area of the clock hand small we stimulate fewer cells for short periods of time. For those reasons, we believed the influence of the clock hand would be negligible. However, it should be considered in future presentations as it might help in explaining some of the inconsistencies found for cardinal axes. Conversely, the idea of similar time courses across pathways found for the lowest contrast appear more natural and is in agreement with other psychophysical and physiological results. Using a contrast cancelation method to measure the intensity of sinusoidal grating afterimages, Kelly and Martinez-Uriegas (Kelly and Martinez-Uriegas, 1993) studied some of the properties of achromatic and chromatic afterimages (MC- and PC-mediated, respectively). Regarding the afterimages formation and how they decay over time, they fitted exponential functions to their data and found no difference between the achromatic and chromatic time constants. They concluded that both types of afterimages had similar temporal properties. On the other hand, physiological records from MC-, PC-, and KC- retinal ganglion cells from the fovea and periphery of primates showed similar time properties using the same time-varying paradigm used here (Zaidi et al., 2012; Bachy and Zaidi, 2014).

The adaptation for simultaneous stimulation of two pathways was slower than adaptation with stimulation of one visual pathway. If pathways did not interact, we would expect the same dynamics in adaptation for non-cardinal responses as those found for cardinal responses, because the independent responses of the three pathways are similar. It is reasonable to expect that the signals coming from RGCs are integrated at a later, likely cortical stage giving rise to a summed signal that drives a shift in the identity point (as our two-stage adaptation model suggested, Figure 6). While higher order chromatic mechanisms have been studied [reviewed by Eskew (2009)], to our knowledge, there are no published results reporting the timing of adaptation for stimuli targeting these mechanisms. Putting aside the parameters, the novelty of the model we proposed is that the shift in adaptation time for non-cardinal stimuli is mathematically accomplished by processing the pooled signal from two pathways with the same mechanism proposed by Zaidi and used in the first stage. Adaptation and the processing of color may have equivalent mechanisms and strategies all along the visual stream (Clifford, 2002; Webster and MacLeod, 2011), so it makes sense for both stages to use the same adaptation process. This is supported by psychophysical results for tasks involving low and high levels of visual processing. For instance, Leopold and colleagues (Leopold et al., 2005) found that the exponential decay of face aftereffects (a high level task) is similar to that found on the aftereffects of orientation (Wolfe, 1984; Magnussen and Johnsen, 1986; Harris and Calvert, 1989) and shape (low levels tasks) (Krauskopf, 1954).

Interestingly, we found adaptation behaved differently for stimuli targeting the same two pathways but with opposite polarity. There is vast literature supporting the theory of multiple mechanisms in which stimuli coming from the same pathways are handled differently (Eskew, 2009). The temporal differences for two intermediate axes in the same plane was modeled by proposing distinct pooling mechanisms for same polarity and for opposite polarity conditions. Although it was not tested, we believe the data could also be modeled with three or more stages, however, this two-stage model seems to be a reasonable representation of physiology as RGCs and the cortex are included. More stages would mean more cortical processing, which is likely, but unnecessary to account for the data.

In summary, the dynamics of adaption with simultaneous stimulation of two visual pathways is different from that when stimulating a single visual pathway. The results can be accounted for by a two-stage adaptation model where the integration of signals from two pathways likely occurs in the cortex.

## Data Availability Statement

The raw data supporting the conclusions of this article will be made available by the authors, without undue reservation.

## Ethics Statement

The studies involving human participants were reviewed and approved by Institutional Review Board of the University of Illinois at Chicago. The patients/participants provided their written informed consent to participate in this study.

## Author Contributions

CP-F and MT worked together setting up experiments, collecting, processing, and analyzing the data as well as writing the manuscript, and accountable for all aspects of the work. DC and SE led this work by helping in all stages of the study, from conception and design to execution, analysis, and manuscript drafting. All authors contributed to the article and approved the submitted version.

## Conflict of Interest

SE was employed by company Peppermill Resort Spa Casino. The remaining authors declare that the research was conducted in the absence of any commercial or financial relationships that could be construed as a potential conflict of interest.

## Publisher’s Note

All claims expressed in this article are solely those of the authors and do not necessarily represent those of their affiliated organizations, or those of the publisher, the editors and the reviewers. Any product that may be evaluated in this article, or claim that may be made by its manufacturer, is not guaranteed or endorsed by the publisher.
